# Three new species of the genus *Araeopteron* Hampson, 1893 (Lepidoptera, Erebidae, Boletobiinae) from the Xizang Autonomous Region, China with an updated list of the world species

**DOI:** 10.3897/zookeys.1060.67674

**Published:** 2021-09-15

**Authors:** Hui Lin Han, Vladimir S. Kononenko

**Affiliations:** 1 School of Forestry, Northeast Forestry University, Harbin, 150040, China; 2 Key Laboratory of Sustainable Forest Ecosystem Management-Ministry of Education, Northeast Forestry University, Harbin, 150040, China; 3 Laboratory of Entomology, Federal Scientific Center of the East Asia Terrestrial Biodiversity, Far Eastern Branch, Russian Academy of Sciences, Vladivostok-22, 690022, Russia

**Keywords:** Araeopteronini, checklist, moths, new species, Noctuoidea systematics, Tibet

## Abstract

Three new species of the genus *Araeopteron* Hampson, 1893: *A.dawai***sp. nov.**, *A.medogensis***sp. nov.** and *A.tibeta***sp. nov.** are described from Motuo (= Medog) County of the Xizang Autonomous Region (= Tibet), China. The imagines as well as the male genitalia are illustrated. A checklist of the 45 species of the genus *Araeopteron* in the world fauna is presented, including recently and presently described species.

## Introduction

The genus *Araeopteron* Hampson, 1893 (type species *A.pictale* Hampson, 1893) belongs to the tribe Araeopteronini, subfamily Boletobiinae, Erebidae. The early authors considered *Araeopteron* in the Eustrotiinae = Erastriinae (Erastrianae sensu Hampson 1910) or Acontiinae (sensu auctorum) of the Noctuidae (Hampson 1910; Inoue 1958, 1965; Nye 1975; Sugi 1982; Poole 1989; Kononenko 1990, 2003, 2005; Kononenko et al. 1998; Fibiger and Agassiz 2001; Fibiger and Hacker 2001).

Araeopteronini Fibiger, 2005 originally has been designated and used as a subfamily Araeopteroninae of the family Noctuidae (Fibiger and Lafontaine 2005; Lafontaine and Fibiger 2006), but later it was downgraded to tribal status and placed to the subfamily Boletobiinae of the family Erebidae (Holloway 2011; Zahiri et al. 2012; Kononenko 2016; Wu et al. 2020).

The group’s distribution is mainly pantropical, but a few species extend into the temperate zone: six species are recorded in Japan, the Russian Far East, and the Korean Peninsula (Inoue 1958, 1965; Kononenko et al. 1998; Kononenko and Han 2007; Fibiger and Kononenko 2008) and one species is recorded in the Near East and southern Europe (Fibiger and Agassiz 2001; Fibiger and Hacker 2001).

The world fauna list of 36 species of *Araeopteron* was published by Fibiger and Kononenko (2008). Subsequently, a review of the Araeopteronini from Borneo followed by a list of species was published by Holloway (2009, 2011). Contributions to the taxonomy of the genus *Araeopteron* was made by Guillermet (2009) and Bippus (2018), designating two new *Araeopteron* species, *A.papaziani* Guillermet, 2009 and *A.legraini* Bippus, 2018 from Réunion Island, western Indian Ocean. The total number of described species of the genus *Araeopteron* in the world fauna (with accounts of the currently described species) now enumerates 45 species.

In China, the genus represented by eight species: *Araeopteronamoena* Inoue, 1958, *A.fragmenta* Inoue, 1965, *A.nebulosa* Inoue, 1965, *A.canescens* (Walker, [1866]), *A.fasciale* (Hampson, 1896), *A.dawai* sp. nov., *A.medogensis* sp. nov., *A.tibeta* sp. nov. distributed from the cool temperate zone to the subtropics.

As a result of intensive collecting and study of the Noctuoidea in remote regions of the Xizang Autonomous Region (= Tibet) in China, three new *Araeopteron* species were found. This article describes and illustrates them in detail.

## Materials and methods

The material was collected by UV light in remote parts of the Xizang Autonomous Region, Tibet, China. Standard methods for dissection and preparation of the genitalia slides have been used as described by Kononenko and Han (2007). Specimens were photographed with a Nikon D700 camera; the genitalia slides were photographed with an Olympus photomicroscope with Helicon Focus software, with the images further processed in Adobe Photoshop CS4.

The materials of the present article, including holotypes, are deposited in the collection of Northeast Forestry University, Harbin, China (**NEFU**).

## Systematic account

### Tribe Araeopteronini

Taxon classificationAnimaliaLepidopteraNoctuidae

Fibiger, 2005

40F8ED88-0683-51AB-9505-5012F91702CB

Araeopteroninae Fibiger, 2005, Esperiana 11: 25 (in Fibiger and Lafontaine 2005). Type genus Araeopteron Hampson, 1893. Lafontaine and Fibiger 2006; Kononenko 2005, 2010; Fibiger and Kononenko 2008; Holloway 2009.
Araeopteronini : Holloway 2011; Zahiri et al. 2012; Kononenko and Pinratana 2013; Kononenko 2016; Wu et al. 2020.

#### Remarks.

The tribe comprises rather uniform and small or very small moths with quadrifine hindwing venation. The most conspicuous autapomorphic character states defining the Araeopteronini are: in external appearance, their small size, and the shape of the wings with a long, narrow, pointed forewing and short, rounded, triangular hindwing; in the male genitalia, the shape of the tegumen, hugely developed paratergal sclerites, the structure of the valve and the articulation of uncus; and in the female genitalia the patch between the ovipositor lobes on the ventral side and the shape of the signum in the corpus bursae (e.g., Fibiger and Hacker 2001; Fibiger and Lafontaine 2005; Fibiger and Kononenko 2008; Holloway 2009).

Araeopteronini is a poorly studied and neglected group of Erebidae moths. At present, the tribe Araeopterinini includes the Old World genus *Araeopteron* with many undescribed species and some other tropical genera belonging to the Boletobiinae (*Hyriodes* Hampson, 1910, *Pseudcraspedia* Hampson, 1889, and *Niaccaba* Walker, 1895) (Holloway 2009, 2011; Kononenko and Pinratana 2013).

### Genus Araeopteron

Taxon classificationAnimaliaLepidopteraNoctuidae

Hampson, 1893

DB7F0504-BA9A-5E96-AEAE-0C9D8FCE2DB8

Figures 1–6 , 7–12

Araeopteron Hampson, 1893, Illustrations of Typical Specimens of LepidopteraHeterocera in the Collection of the British Museum 9: 33, 136. Type species: Araeopteronpictale Hampson, 1893 [Sri Lanka].

#### Synonymy.

*Araeopterum* Hampson, 1896, emendation; *Thelxinoa* Turner, 1902; *Essonistis* Meyrick, 1902; *Araeopterella* Fibiger & Hacker, 2001; *Araeoptera* Hampson, 1910, emendation.

#### References.

Inoue 1958, 1965; Nye 1975; Sugi 1982; Poole 1989; Kononenko 1990, 2003, 2005, 2010, 2016; Kononenko et al. 1998; Fibiger and Agassiz 2001; Fibiger and Hacker 2001; Fibiger 2002; Kononenko and Han 2007; Fibiger and Kononenko 2008; Guillermet 2009; Holloway 2009, 2011; Kononenko and Pinratana 2013; Bippus 2018; Wu et al. 2020.

#### Diagnosis.

Small and very small species, wingspan 9–18 mm. Forewing narrow, with oblique outer margin and long fringes; hindwing shorter than forewing, with shallow concavity under apex; wing colour grey or brown-grey, in some species with orange or pale reddish patches, reniform stigma black; frons scaled. In the male genitalia, tegumen short, broad, paratergal sclerites uniting the tegumen and vinculum hugely developed; vinculum short and broad; uncus with long coecum; costa and cucullus membranous; sacculus sclerotised, narrow; apex of sacculus spatulate or club-shaped; uncus thin, rather short, curved. In the female genitalia, a small raised membranous or slightly sclerotised patch or low cone covered with long hair-like setae lies between posterior ends of anal papillae; signum cone-like or hat-like with a rounded top, fringed basally with spines; sometimes signum as relatively large flat plate. Larva and food specialisation are unknown.

**Figures 1–6.**
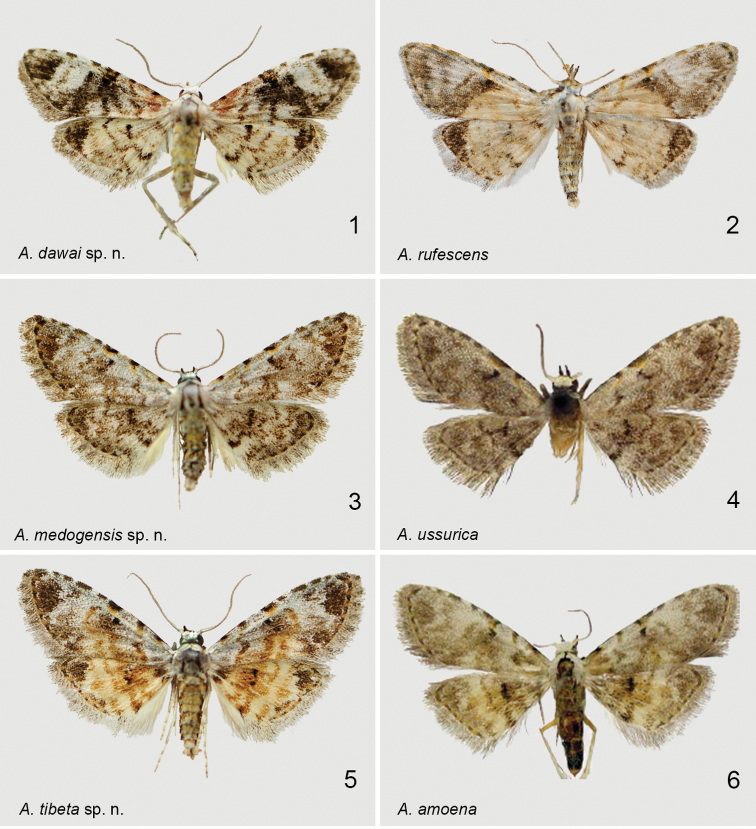
Adults of *Araeopteron* spp. **1***A.dawai* sp. nov. holotype **2***A.rufescens*. Malaysia, Borneo **3***A.medogensis* sp. nov. holotype **4***A.ussurica* (Russia, Primorye, after Fibiger and Kononenko 2008) **5***A.tibeta* sp. nov. holotype **6***A.amoena* (Russia, Primorye, after Fibiger and Kononenko 2008).

The genus includes 45 described species and many undescribed species distributed mainly in tropical and subtropical regions; a few species extend into the temperate zone.

In China five described species of the genus *Araeopteron* are known (Fibiger and Kononenko 2008), of which two species, *A.canescens* (Walker, [1866]) and *A.fasciale* (Hampson, 1896) have recently been found in southeast China by Wu et al. (2020). Three further new *Araeopteron* species are described below.

### Araeopteron dawai sp. nov.

Taxon classificationAnimaliaLepidopteraNoctuidae

5148447C-E71E-5B5A-8B6A-15F694B79CBF

http://zoobank.org/E70C4C1C-18F5-46B0-B103-77E5F0FF8574

**Figures 7–12.**
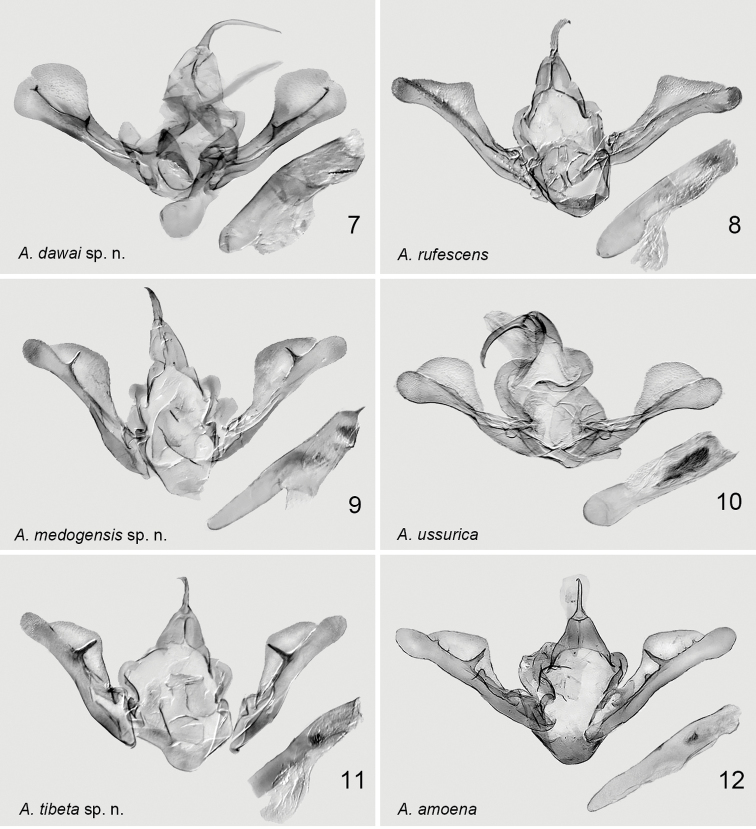
Male genitalia of *Araeopteron* spp. **7***A.dawai* sp. nov. holotype, gen. prep. no. hhl-4010-1 **8***A.rufescens*, Malaysia, Borneo, gen. prep. no. hhl-4587-1 **9***A.medogensis* sp. nov. holotype, gen. prep. no. hhl-4022-1 **10***A.ussurica* (Russia, Primorye, after Fibiger and Kononenko 2008) **11***A.tibeta* sp. nov. holotype, gen. prep. no. hhl-4026-1 **12***A.amoena* (Russia, Primorye, after Fibiger and Kononenko 2008).

**Figures 13–15.**
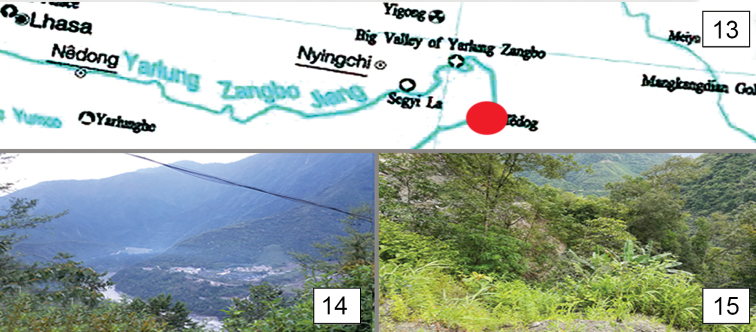
The map (**13**) and habitat (**14, 15**) of Araeopteron spp. in the Xizang Autonomous Region, China

Figures 1 , 7 , 13–15

#### Type material.

***Holotype*** male, China, Xizang Autonomous Region, Motuo (= Medog) County, 16–17.iv.2018, H.L. Han, genit. prep. no. hhl-4010-1 (NEFU). ***Paratypes*.** 2 ♂♂, same data as holotype, genit. prep. no. hhl-4009-1 (NEFU).

#### Diagnosis.

The new species (Figs 1, 7), externally and in the male genitalia, is similar to *A.rufescens* Hampson, 1910 (Sri Lanka, Malaysia, Borneo; figs 2, 8), but differs by a narrower forewing with sharp apex, bearing a dark triangular patch (in *A.rufescens* apex blunt, without blackish triangular patch; only a weak arched dark band present); the transverse lines are distinct (in *A.rufescens* they are indistinct); the dark apical triangular patch on the hindwing is small (in *A.rufescens* it is broader); the discal spot is distinct and stout (in *A.rufescens* indistinct and slender).

***Male genitalia***: clasper with medially sclerotised harpe and small thorn-like apical extension (in *A.rufescens* the clasper with small smooth teeth apically); the costa rounded and swollen in the terminal part of the valva (in *A.rufescens* it is swollen and triangular in apical third of valva); the uncus as long as the tegumen (in *A.rufescens* the uncus is ca 1/2 tegumen length); aedeagus slightly curved (in *A.rufescens* it is straight); vesica with a toothed band (in *A.rufescens* it bears more than 20 small thin spines).

#### Description.

Adult (Fig. 1). Wingspan 11.5–12.0 mm. Antennae filiform, head, patagia, and tegulae covered with flat white scales, thorax whitish with grey; abdomen greenish yellow, mixed with white. Forewing pale yellow to pale greyish yellow, mixed with a little orange; apex rather sharp; basal area dark orange, basal line expressed with distinct black costal dot; antemedial line blackish brown, almost black, wavy, oblique; median line double, black, filled with mixed brown with orange inside, smoothly incurved, with pale black and orange patches between lines; postmedial line brownish orange and strongly arched before Cu_2_, its other part mixed with black, and incurved to inner margin; subterminal line brownish black at costal margin, other part fused with blackish apical patch; terminal line brown to blackish brown, with black dots at the tops of veins; reniform stigma dark black; apex with large black triangular patch; basal, antemedial, and median areas densely covered with orange; postmedial area pale to greyish white, with blackish brown to brown at inner margin; subterminal area pale greyish; fringe grey, mixed with brown; pale and greyish parts of the postmedial and subterminal area forming large patch. Hindwing pale greyish yellow to faint yellow; antemedial line smoky-brown to brownish black, wavy; median line orange, weakly waved; postmedial line brown to brownish black, wavy, incurved posteriorly; subterminal line smoky orange, indistinct; apex sharp with single large black triangular patch; fringe thin and lighter than in forewing; discal spot dark black, formed by two dots.

***Male genitalia*** (Fig. 7). Tegumen triangular, as narrow, strongly arched band; paratergal sclerits hugely developed, flat, curved; vinculum, thick, sclerotised. Saccus U-shaped. Valva racket-shaped, narrower medially, extended and rounded apically; sacculus thin, gradually narrower to 3/4 length of valva, then broadened and rounded apically; clasper fused to sacculus, with pointed triangular ampullae medially and small, hook-like apical extension; costa sclerotised, thick basally, gradually extended and membranous apically; cucullus large with small medial incurving on inner margin. Uncus thin, relatively long (as long as tegumen), smoothly curved medially, sclerotised. Juxta plate-like, large, rounded, slightly sclerotised. Aedeagus short, cylindrical, slightly curved, weakly sclerotised posteriorly; coecum short, ca 1/4 as long as aedeagus; vesica with sclerotised band of teeth.

***Female genitalia***. Female unknown.

#### Distribution.

(Fig. 13). The species is known only from its type locality: China, Xizang Autonomous Region (Tibet), Motuo (= Medog) County.

#### Etymology.

The species name is dedicated to Mr Wa Da, Chinese entomologist, a famous insect researcher of the fauna in the Xizang Autonomous Region, China.

#### Bionomics.

(Figs 14, 15). The new species was collected in Motuo County, Xizang in April 2018 in the intermediate zone between the subtropical rain forest and broad-leaf forest zones, at an altitude 1121 m.

### Araeopteron medogensis sp. nov.

Taxon classificationAnimaliaLepidopteraNoctuidae

BCF70843-144A-5DB3-B78B-1590BBBE8BE9

http://zoobank.org/E19FDE59-FC6E-4009-B895-B922B4BFBD91

Figures 3 , 9 , 13–15

#### Type material.

***Holotype*** male, China, Xizang Autonomous Region, Motuo (= Medog) County, 16–17.iv.2018, H.L. Han, genit. prep. no. hhl-4022-1 (NEFU). ***Paratypes*.** 6 ♂♂, same data as holotype, genit. prep. nos. hhl-4021-1, hhl-4023-1, hhl-4024-1, hhl-4025-1 (NEFU).

#### Diagnosis.

The new species, superficially and by the structure of the male genitalia, is similar to *A.ussurica* Fibiger & Kononenko, 2008 (Figs 4, 10), but can by separated from it by the following characters: the basal line present only as a black dot at the costal margin (in *A.ussurica* the basal line is absent); the transverse lines in the costal margin mixed greyish yellow colour (in *A.ussurica* the transverse lines in the costal margin greenish grey); the terminal area coloured with smoky-brown to black (in *A.ussurica* smoky but the terminal area is grey); the wing ground colour in the new species compared with *A.ussurica* is more whitish (in *A.ussurica* it is darker greyish); the wing pattern of the new species is more distinct with stronger colour contrast, compared with *A.ussurica*, in which the ground colour is pale greyish. In the male genitalia, the paratergal sclerite is moderate in length and slightly rounded (in *A.ussurica* it is huge and strongly rounded) ; vinculum narrower (in *A.ussurica* it is much broader); saccus U-shaped (in *A.ussurica* it is V-shaped); the harpe is needle-like, placed in the apical part of valva, ca 3/4 length of valva from its basal part (in *A.ussurica* harpe as a small bulge 2/3 from basal part of valva); the costa with a smoothly arched bulge in apical 1/2 of valva (in *A.ussurica* the costa with round bulge); the uncus short, as long as 1/2 of tegumen, slightly curved apically vs. 2X longer than tegumen, hooked apically uncus in *A.ussurica*; the aedeagus is narrow and long vs. short and broad in *A.ussurica*; the carina with spines (in *A.ussurica* the carina without spines); the vesica with two sclerotised cornute patches (in *A.ussurica* the vesica with a large cornute band formed by numerous thin, small spines).

#### Description.

**Adult** (Fig. 3). Wingspan 11.0–12.5 mm. Antennae filiform. Head, patagia, tegulae, and thorax covered with white scales; abdomen greyish white, mixed with orange. Forewing pale greyish yellow, mixed with brown; forewing apex blunt, rounded; basal line present as a black dot at costal margin, its other part distinct, grey with small yellow scales; antemedial line oblique, thin, wavy, brown with yellow at costal area; median line double, indistinct, thin, wavy, smoky-brown between double lines, and as distinct black dot at costal margin; postmedial line broad, brown, rising to M_3_, slightly curved, then bending and going obliquely to inner wing margin; subterminal line as brownish green dots at costal margin, its other part fused to brownish apical patch; terminal line pale brown to brownish green, with black dots on vein; reniform stigma dark, black, formed by two diffused dots; terminal area pale brown to brownish green; fringe grey, mixed with brown, in basal part with yellow. Hindwing pale greyish with white, slightly darker than forewing; antemedial line smoky-brown, indistinct; median line blackish brown, indistinct, weakly waved; postmedial line slender, brown to brownish black, wavy, sharp at Cu_1_ area; subterminal line smoky-brown, slightly mixed with black, wavy, indistinct; terminal line and fringes same as on forewing; discal spot prominent, dark brown, slightly diffused.

***Male genitalia*** (Fig. 9). Tegumen triangular, narrow dorsally. Paratergal sclerits thin, broaded apically. Vinculum thick, sclerotised, U-shaped, flat, and broader posteriorly. Saccus U-shaped, weakly sclerotised. Valva constricted basally; sacculus thin, sclerotised, gradually narrower medially, then gradually broader and rounded apically, exceed cucullus; clasper fused to sacculus, with pointed and tapered harpe in its apical third; costa slightly sclerotised, with minute grains, thin basally, gradually arched and swollen apically. Uncus relatively short and solid, ca 1/3 length of tegumen, slightly curved, sclerotised. Juxta large, plate-like, with bulb at centre, and broad outer frame. Aedeagus long, cylindrical, slightly curved, carina with short spines, slightly sclerotised; coecum as long as 1/2 length of aedeagus; vesica with weakly sclerotised grainy band and plate.

***Female genitalia***. Female unknown.

#### Distribution.

(Fig. 13). The species is known only from its type locality: China, Xizang Autonomous Region (Tibet), Motuo (= Medog) County.

#### Etymology.

The species name refers to the Tibetan name of the type locality Medog in Tibet, China.

#### Bionomics.

(Figs 14, 15). The new species has been collected in Motuo County of Xizang in April in the intermediate zone between subtropical rain forest and broad-leaf forest zones, at an altitude 1121 m.

### Araeopteron tibeta sp. nov.

Taxon classificationAnimaliaLepidopteraNoctuidae

580CDD45-3807-507B-8511-999472392364

http://zoobank.org/2E7076ED-9044-42E5-9FA9-46BB2FA9FA76

Figures 5 , 11 , 13–15

#### Material examined.

***Holotype***: male, China, Xizang Autonomous Region, Motuo (= Medog) County, 16–17.iv.2018, H.L. Han, genit. prep. no. hhl-4026-1 (NEFU). ***Paratype***: 1 male with same data (NEFU).

#### Diagnosis.

The new species is similar to *A.amoena* (Figs 6, 12, 13–15) by the external appearance and the male genitalia but differs by the more colourful and distinct wing pattern with greyish orange medial part of the forewing and orange for most of the hindwing (in *A.amoena* the wing pattern and colouration is less distinct and less colourful, with a greyish orange patch in the middle of the hindwing); the forewing basal line in *A.tibeta* presents as a black dot on the costal margin and continues as a very thin line (in *A.amoena* only the small black dot at the costal margin is expressed); blackish apical patch distinct (in *A.amoena* it is indistinct); transverse lines of the forewing distinct (in *A.amoena* they are indistinct); reniform stigma streak-like, formed with two dots (in *A.amoena* it presents as a small dot); the hindwing with black triangular apical patch (in *A.amoena* the triangular apical patch is diffused; fringe paler than in *A.amoena*). In the male genitalia, clasper with spine-like harpe, without three small tooth-like extensions present in *A.amoena*; the smoothly arched bulge of costa is rather straight posteriorly, slightly constricted anteriorly (in *A.amoena* it is smooth and slightly curved posteriorly).

#### Description.

Adult (Fig. 5). Wingspan 11.5–12.0 mm. Antennae filiform. Head, patagia, and tegulae covered with white scales; thorax greyish white, with two medial patches of blackish scales. Abdomen greenish brown, mixed with orange. Forewing pale greyish white; apex rather sharp; basal line present only as a black dot at costal margin, its other part diffused, slender; antemedial line brown, wavy, oblique, with brownish yellow patch at costal area; median line double, reddish brown, diffused, wavy, greyish brown between lines, and as distinct blackish dot at costal margin; postmedial line brown, indistinct, wavy, with fracture at costal region; subterminal line only as brown dot at costal margin, in other part fused to apical greyish brown patch; terminal line pale brown to greenish brown, with black dot at vein; reniform stigma dark black, formed by two fused dots; terminal area blackish brown to dark brownish, forms ovoid patch at wing apex; medial part of wing greyish orange brown colouration; fringe grey, mixed with blackish brown, yellow basally. Hindwing greyish orange-yellow; antemedial line blackish brown, broad and distinct; median line reddish brown, indistinct; postmedial line thin, reddish brown, slightly waved, with blackish inner border; subterminal line reddish brown, slightly waved; terminal area, terminal line, and fringe same colour as on forewing; discal spot dark black, formed with two dots.

***Male genitalia*** (Fig. 11). Tegumen triangular posteriorly, thick, curved, broad anteriorly. Paratergal sclerits large, but smaller than in related *A.amoena*. Vinculum thick, sclerotised, flat, U-shaped, broad band-like posteriorly. Saccus U-shaped, membranous. Valva constricted at 1/3 from base; sacculus thick, sclerotised, gradually constricted to broad medial part, rounded apically, and exceeding cucullus; clasper fused to sacculus, with pointed, sclerotised tapered harpe, placed ca 3/5 length from base of valva; costa slightly sclerotised, minutely granulated, thin basally, gradually arched and extended to swollen, membranous middle part. Uncus short, as long as 1/2 of tegumen, thin, slightly curved apically. Juxta large, plate-like sclerotisation posteriorly. Aedeagus long, cylindrical, slightly curved, weakly sclerotised, carina with very short spines; coecum as long as ca 1/2 aedeagus; vesica with weakly sclerotised patch of minute spines medially.

***Female genitalia***. Female unknown.

#### Distribution.

(Fig. 13). The species is known only from its type locality: China, Xizang Autonomous Region (Tibet), Motuo (= Medog) County.

#### Etymology.

The species name refers to Tibet.

#### Bionomics.

(Figs 14, 15). The new species has been collected in Motuo County of Xizang in April in the intermediate zone between subtropical rain forest and broad-leaf forest zones, at an altitude of 1121 m.

##### Checklist of the genus *Araeopteron* Hampson, 1893 of the world

***Araeopteronacidalica*** (Hampson, 1910), “Catalogue of the Lepidoptera Phalaenae in the British Museum” 10: 22, fig. 9 (*Araeoptera*). Type locality: Jamaica, Moneagaue.

***Araeopteronadeni*** Fibiger & Hacker, 2001, “Esperiana” 8: 578, Pl. 28: 8. Type locality: Yemen, Prov. Abyan, 50 km NE Aden, 7 km NNW Zinjibar, 50 m.

***Araeopteronalboniger*** Fibiger & Hacker, 2001, “Esperiana” 8: 581, Pl. 28: 14, 15. Type locality: Yemen, Prov. Ibb, Lower Wadi Duur, village Azuhirya, 1300 m.

***Araeopteronamoena*** Inoue, 1958, “Tinea” 4: 230, fig. 2. Type locality: Japan, Kangawa Pref., Chigasaki.

***Araeopteronaulombardi*** Fibiger & Hacker, 2001, “Esperiana” 8: 580, Pl. 28: 12, 13. Type locality: Yemen, Prov. Ibb, Wadi Merhab, village Lajajil, 1600 m.

***Araeopteronbetie*** (Dyar, 1914), “Procceedings of the United States National Museum” 47: 184 (*Araeoptera*). Type locality: Panama, Trinidad.

***Araeopteroncanescens*** (Walker, 1866), “Illustrations of Typical Specimens of LepidopteraHeterocera in the Collection of the British Museum” 34: 1318 (?*Isopteryx*). Type locality: Australia, Queensland, Moreton Bay.

= *favillalis* (Walker, [1866]), “Illustrations of Typical specimens of LepidopteraHeterocera in the Collection of the British Museum” 34: 1319 (?*Isopteryx*). Type locality: Australia, Queensland, Moreton Bay.

***Araeopterondawai*** Han & Kononenko, sp. nov., “ZooKeys” (present publication). Type locality: China, Xizang Autonomous Region, Motuo County.

***Araeopterondiehli*** Fibiger, 2002, “Heterocera Sumatrana” 12.3: 129. Type locality: Sumatra, S Medan, Dolok Merengir (*Simarsopa*) 170 m.

***Araeopteronecphaea*** (Hampson, 1914), “Annals and Magazine of Natural History” (8)13: 167 (*Araeoptera*). Type locality: Nigeria, Faro.

***Araeopteronelam*** (Schaus, 1911), “Annals and Magazine of Natural History” (8)8: 108 (*Acidaliodes*). Type locality: Costa Rica, Juan Vinas.

***Araeopteronepiphracta*** (Turner, 1902), “Proceedings of the Linnaean Society of New South Wales” 27: 132 (*Thelxinoa*). Type locality: Australia, Queensland, Brisbane.

***Araeopteronfasciale*** (Hampson, 1896), “The Fauna of British India, Including Ceylon and Burma. Moths” 4: 543 (*Araeopterum*). Type locality: Sri Lanka.

***Araeopteronflaccida*** Inoue, 1958, “Tinea” 4: 229, Pl. 32: 1. Type locality: Japan, Kanagawa Pref., Chigasaki.

***Araeopteronfragmenta*** Inoue, 1965, “Tinea” 7: 81, Pl. 15: 5A, 5B. Type locality: Japan, Kanagawa Pref., Fujisawa.

***Araeopterongoniophora*** Hampson, 1907, “Journal of the Bombay Natural History Society” 17: 670. Type locality: Sri Lanka, Nawalpitiya.

***Araeopterongriseata*** Hampson, 1907, “Journal of the Bombay Natural History Society” 17: 670. Type locality: Sri Lanka.

***Araeopteronimbecilla*** (Turner, 1933), “Transactions and Proceedings of the Royal Society of South Australia” 57: 161 (*Araeoptera*). Type locality: Australia, North Queensland, Babinda.

***Araeopteronkoreana*** Fibiger & Kononenko 2008, "Zootaxa" 1891: 50, Figs 11, 18. Type locality: South Korea, Pyounchang GW, Moonsen-ri.

***Araeopteronkurokoi*** Inoue, 1958, “Tinea” 4: 230. Type locality: Japan, Fukoka Pref., Hikosan Mt.

***Araeopteronlegraini*** Bippus, 2018; “Phelsuma” 26: 23; Type locality: Réunion, La Possession, 400 m.

***Araeopteronleucoplaga*** (Hampson, 1910), “Catalogue of the Lepidoptera Phalaenae in the British Museum” 10: 29, Pl. 149: 19 (*Araeoptera*). Type locality: Borneo, Pulo Laut.

***Araeopteronmakikoae*** Fibiger & Kononenko 2008, "Zootaxa" 1891: 49, Figs 7, 8, 16, 24, 29. Type locality: Russia, Primorye terr., Gornotaezhnoe.

***Araeopteronmedogensis*** Han & Kononenko, sp. nov., “ZooKeys” (present publication). Type locality: China, Xizang Autonomous Region, Motuo County.

***Araeopteronmicraeola*** (Meyrick, 1902, April), “Transactions of the Entomological Society of London” 35: 36 (*Essonistis*). Type locality: Australia, Queensland, Brisbane.

= *calliscia* Turner, 1902 (*Thelxinoa*), “Proceedings of the Linnaean Society of New South Wales”. Type locality: Australia, Queensland, Brisbane.

***Araeopteronmicroclyta*** (Turner, 1920), “Transactions and Proceedings of the Royal Society of South Australia” 44: 161 (*Araeoptera*). Type locality: Australia, North Queensland, Kuranda.

***Araeopteronminimale*** Freyer, 1912, “Transactions of the Linnaean Society of London (Zool.)” 15(1): 11. Type locality: Seychelles, Mahe.

***Araeopteronnebulosa*** Inoue, 1965, “Tinea” 7: 82, pl, 15: 4A, 4B. Type locality: Japan, Shizouka Pref., Odaru Spa.

***Araeopteronnivalis*** Hampson, 1907, “Journal of the Bombay Natural History Society” 17: 671. Type locality: Sri Lanka, Paradeniya.

***Araeopteronobliquifascia*** (Joanis, 1910), “Bulletin de la Société Entomologique de France” 1910: 201 (*Araeoptera*). Type locality: Mauritius, Curepipa.

***Araeopteronpapaziani*** Guillermet, 2009; “L’Entomologiste” 65 (3): 121; Type locality: Réunion, Les Avirons 250 m.

***Araeopteronpictale*** Hampson, 1893, “Illustrations of Typical Specimens of LepidopteraHeterocera in the Collection of the British Museum” 9: 33, 137, Pl. 168: 19. Type locality: Sri Lanka, Pundaloya.

***Araeopteronpleurotypa*** (Turner, 1902), “Proceedings of the Linnaean Society of New South Wales” 27: 133 (*Thelxinoa*). Type locality: Australia, Queensland, Cairns, Townsville.

***Araeopteronpoliobapta*** (Turner, 1925), “Transactions and Proceedings of the Royal Society of South Australia” 44: 39 (*Araeoptera*). Type locality: Australia, Queensland, Montville.

***Araeopteronpoliophaea*** (Hampson, 1910), “Catalogue of the Lepidoptera Phalaenae in the British Museum” 10: 29, Pl. 149: 20 (*Araeoptera*). Type locality: Sri Lanka, Maskeliya.

***Araeopteronproleuca*** Hampson, 1907, “Journal of the Bombay Natural History Society” 17: 671. Type locality: India, Sri Lanka.

***Araeopteronrufescens*** (Hampson, 1910), “Catalogue of the Lepidoptera Phalaenae in the British Museum” 10: 27, Pl. 149: 17 (*Araeoptera*). Type locality: Sri Lanka, Kegalle.

***Araeopteronschreieri*** Fibiger & Hacker, 2001, “Esperiana” 8: 579, Pl. 28: 10, 11. Type locality: Yemen, 36, Prov. Al Hudaydah, Jabal Burra, 25 km SE Bajil, 600 m.

***Araeopteronsterrhaoides*** (Fibiger & Hacker, 2001), “Esperiana” 8: 582: Pl. 28: 16, 17 (*Araeopterella*). Type locality: Yemen, Prov. Sanaa, Jabal Raymah, 25 km E Al Mansuriyah, Wadi Bullbull, 2 km SE Khansa, 700 m.

***Araeopterontibeta*** Han & Kononenko, sp. nov., “ZooKeys” (present publication). Type locality: China, Xizang Autonomous Region, Motuo County.

***Araeopteronvilhelmina*** (Dyar, 1916), “Proceedings of the United States National Museum” 51: 18 (*Araeoptera*). Type locality: Mexico, Tabasco, Teapa.

***Araeopteronxanthopis*** Hampson, 1907, “Journal of the Bombay Natural History Society” 17: 672. Type locality: Sri Lanka, Haldamulla.

***Araeopteronyemeni*** Fibiger & Hacker, 2001, “Esperiana” 8: 577, Pl. 8: 4. Type locality: Yemen, Prov. Sanaa, Jabal Raymah, 25 km E Al Mansuriyah, Wadi Bullbull, 2 km SE Khansa, 700 m.

## Supplementary Material

XML Treatment for Araeopteronini

XML Treatment for Araeopteron

XML Treatment for Araeopteron dawai

XML Treatment for Araeopteron medogensis

XML Treatment for Araeopteron tibeta
